# Adaptive platform trials using multi-arm, multi-stage protocols: getting fast answers in pandemic settings

**DOI:** 10.12688/f1000research.26253.2

**Published:** 2020-11-23

**Authors:** Nurulamin M. Noor, Sarah L. Pett, Hanif Esmail, Angela M. Crook, Claire L. Vale, Matthew R. Sydes, Mahesh K.B. Parmar

**Affiliations:** 1 Medical Research Council Clinical Trials Unit, University College London, London, WC1V6LJ, UK

**Keywords:** multi-arm, multi-stage, MAMS, adaptive, platform, trials, efficient, conduct, epidemic, pandemic, SARS-CoV-2, COVID-19, FAME, IPD, meta-analysis

## Abstract

Global health pandemics, such as coronavirus disease 2019 (COVID-19), require efficient and well-conducted trials to determine effective interventions, such as treatments and vaccinations. Early work focused on rapid sequencing of severe acute respiratory syndrome coronavirus 2 (SARS-CoV-2), subsequent *in-vitro* and *in-silico *work, along with greater understanding of the different clinical phases of the infection, have helped identify a catalogue of potential therapeutic agents requiring assessment.

In a pandemic, there is a need to quickly identify efficacious treatments, and reject those that are non-beneficial or even harmful, using randomised clinical trials. Whilst each potential treatment could be investigated across multiple, separate, competing two-arm trials, this is a very inefficient process. Despite the very large numbers of interventional trials for COVID-19, the vast majority have not used efficient trial designs.

Well conducted, adaptive platform trials utilising a multi-arm multi-stage (MAMS) approach provide a solution to overcome limitations of traditional designs. The multi-arm element allows multiple different treatments to be investigated simultaneously against a shared, standard-of-care control arm. The multi-stage element uses interim analyses to assess accumulating data from the trial and ensure that only treatments showing promise continue to recruitment during the next stage of the trial.

The ability to test many treatments at once and drop insufficiently active interventions significantly speeds up the rate at which answers can be achieved. This article provides an overview of the benefits of MAMS designs and successes of trials, which have used this approach to COVID-19. We also discuss international collaboration between trial teams, including prospective agreement to synthesise trial results, and identify the most effective interventions. We believe that international collaboration will help provide faster answers for patients, clinicians, and health care systems around the world, including for each further wave of COVID-19, and enable preparedness for future global health pandemics.

## Introduction

Global health pandemics pose many challenges, which require rapid clinical answers. There have been a growing number of global health emergencies caused by infectious diseases, including the novel coronaviruses, such as severe acute respiratory syndrome coronavirus 2 (SARS-CoV-2) ^1^, and recent outbreaks of Ebola and Zika ^2^. These have all been associated with significant morbidity and mortality, and demanded urgent evaluation for treatments and development of vaccines. With increasing globalisation, similar challenges are likely to be encountered with further waves of these diseases, and further novel infectious pathogens entering the human population. There is therefore a need for more efficient, well-conducted, and collaborative clinical trials to provide answers for such pandemic settings.

From the outset of the coronavirus disease 2019 (COVID-19) pandemic, there has been an astounding pace of discovery to characterise this condition. Within months after the first widely-reported cases, the genomic structure of SARS-CoV-2 causing COVID-19 was identified and work initiated to explore downstream biology ^3^. Accordingly, there are multiple potential interventions with plausible biological mechanisms that have been proposed and are under investigation for treatment or prevention of COVID-19 ^4, 5^.

## Urgent need for clinical trial efficiency in a pandemic setting

SARS-CoV-2 exemplifies the typical challenges of highly infectious pathogens entering the human population for the first time, given that there were initially no approved treatments nor any available vaccines. There was a need for answers even more quickly from clinical trials. Both a need to identify effective treatments for those most vulnerable to COVID-19 and, equally important, to identify ineffective interventions early, in order to direct resources to the most promising interventions.

Researchers are generally aware of the potential biases from non-randomised trials and small, underpowered randomised controlled trials (RCTs) ^6^, as well as the difficulties of interpreting non-controlled cohort studies ^7^. However in a pandemic setting, particularly for a condition with high risks of morbidity and mortality, there is typically demand for early access to treatments with potential benefit - even in the absence of any compelling RCT data ^8^.

A review by the National Academy of Science, Engineering and Medicine from the United States of America (USA), on the international response to the 2014 Ebola outbreak in West Africa, highlighted many apparent errors and lessons to be learned ^9^. A key criticism was that small, underpowered clinical trials did not provide answers to help direct clinical care, nor did “compassionate use” trial programmes, since even if treatments were provided to large numbers of individuals, little sense of relative efficacy could be made in the absence of a comparative control arm ^10^. Indeed, there was a widespread recognition of the need for randomisation to determine if interventions are effective or not in global health pandemics.

## Efficiencies of MAMS platform designs to investigate multiple interventions

Each potential intervention for COVID-19 could be investigated across separate traditional two-arm, RCTs. This is, however, an inefficient process, particularly if, as expected, a large number of these interventions turn out to be clinically ineffective.

Unfortunately, the lessons from previous epidemics have not been widely applied to COVID-19. The use of efficient trial approaches have been the exception rather than the rule, with over 2,500 separate clinical trials registered for COVID-19 by 25 August 2020 on the World Health Organisation (WHO) International Clinical Trials Registry Platform (ICTRP) ^11^. A large number of these registrations are for small, underpowered trials using traditional non-adaptive designs, and this inefficiency has been compounded by many overlapping and competing trials seeking to answer the same clinical question ^12^.

Moreover, the inefficiencies of using multiple, classical, two-arm RCTs have been felt even more keenly during the COVID-19 pandemic, given the ability of such an infectious pathogen to rapidly overwhelm health service capacity. First, each two-arm trial has a separate control group, which is hugely inefficient given the large overall numbers of patients required; second, the choice of interventions to be tested is often based on very limited information, which will inevitably lead to a large number of ‘negative’ trials. Finally, as information emerges, further interventions demand evaluation immediately and setting up a separate, new trial is again hugely inefficient and time consuming.

There has been a growing recognition of the need to adopt efficient RCT approaches in pandemic settings ^13^. Indeed, irrespective of the medical condition, whenever there are multiple interventions meriting further assessment, there has increasingly been a cultural shift away from two-arm RCTs, towards multi-arm designs ^14^. A multi-arm, multi-stage (MAMS) platform design, provides a solution to many of the problems described and could be considered for evaluation of treatments or preventative strategies.

The multi-arm element allows multiple different interventions (comparisons) to be investigated simultaneously against a shared, standard-of-care control arm ^15^. The shared control arm approach means that fewer patients are required overall compared with numerous, separate trials and allows for greater assignment of participants to receive comparison interventions.

The multi-stage element of a MAMS design uses interim analyses to assess accumulating data from the trial and ensure that only treatments showing promise continue to recruit new participants during the next stage of the trial. Whilst therapies showing a lack of sufficient benefit or, indeed, signals of real harm, have recruitment halted.

The ability to test many interventions at once and move recruitment away from insufficiently effective interventions, significantly speeds up the rate at which answers can be achieved ^16^. The design also ensures resource and funding are allocated to the most promising arms. Importantly, the MAMS platform allows new interventions to be rapidly added for assessment at any time, via an approved protocol amendment rather than by launching a new or competing trial. The selection of new intervention arms is a challenging decision in a pandemic if there is a large number of interventions suitable for evaluation and limited information about both the disease and potential interventions. Ultimately selection of intervention arms are the responsibility of the trial team and made based on a range of information, including promising data from laboratory, animal or human studies, as well as consideration of factors such as cost and wider availability of the intervention ^16^.

## Advantages of MAMS platform design in non-pandemic settings

The MAMS platform has been successfully implemented in non-pandemic settings. Whilst adaptive, platform approaches have increasingly been used across early-phase trials for drug screening programmes in industry ^17^, uptake in the late-phase setting has been more gradual ^18^.

The MAMS approach was perhaps first designed and applied in the late-phase setting to ovarian cancer in the ICON5 trial (NCT00011986) ^19, 20^, where many design features were implemented for the first time, and numerous lessons learned were incorporated into the STAMPEDE trial (NCT00268476) ^21^. STAMPEDE has utilised a MAMS platform approach to investigate potential treatments for prostate cancer. This trial has been running since 2005 and will address at least ten randomised treatment comparisons over 20 years (by 2025). In the past 15 years, results from STAMPEDE have contributed to three changes in standard-of-care for patients with prostate cancer ^22^. Separate parallel-group, two-arm trials would have taken many decades to produce such results.

The MAMs platform approach changes the standard research question from a two-arm RCT of ‘does this intervention improve outcomes?’, to the more informative question ‘do any of these interventions, and any further new interventions identified, which need to be tested, improve outcomes?’. Given the need for speed and the large proportion of RCTs that do not show a new intervention is better than the control, we believe that this is a more relevant research question to ask – and potentially offers great utility for infectious diseases in pandemic settings. 

## Use of a MAMS platform approach for infectious disease pandemics

In recent years, it has been shown that the MAMS approach can also be applied in the fast-moving context of global health emergencies, and equally within resource-limited healthcare settings.

In the 2016 Ebola outbreak in West Africa, four potential treatments for Ebola were simultaneously investigated in the PALM trial (NCT03719586) ^23^, with two treatments being “dropped” following interim analysis and the two more effective treatments proceeding to a further next stage of recruitment ^23^. The PALM consortium demonstrated the ability of such modern RCTs to be rigorously conducted during infectious disease outbreaks and importantly to deliver clinical answers in a timely fashion.

Many treatments and trials have been proposed and initiated for COVID-19, and the extensive, ongoing screening projects of new and re-purposed agents will likely lead to many more potential interventions that require evaluation in the near future. Commendable private-public collaborations, such as the Accelerating COVID-19 Therapeutic Interventions and Vaccines (ACTIV), programme have been setup to develop and test interventions at much greater speed than typical development programmes ^24^. Using a MAMS platform design offers a very efficient way to assess these interventions in RCTs, in order to make progress as rapidly as possible ^25^.

Only a small number of trials have utilised a MAMS platform approach for COVID-19. However, the benefits of a co-ordinated and collaborative approach using an efficient platform design, are well illustrated by the RECOVERY trial (NCT04381936). RECOVERY, being co-ordinated from the United Kingdom (UK), initially started as a four-arm trial, with multiple further comparisons added to date. Recruitment was able to start within nine days of the protocol being finalised ^26^, and remarkably three research questions were answered - with over 12,000 participants recruited - within a period of just over 100 days. Most importantly this trial has provided convincing results to influence changes in clinical practice ^27– 29^.

There are a number of further notable trials using a MAMS platform approach for patients with COVID-19. The SOLIDARITY master protocol (ISRCTN83971151) advocated and co-ordinated by the WHO and adopted across many individual countries. The ACTT-3 trial (NCT04492475) -- an international trial co-ordinated from the National Institute of Allergy and Infectious Diseases in the USA, initially starting as a three-arm trial. This builds on successes of the adaptive two-arm ACTT-1 trial (NCT04280705), which demonstrated improved time to recovery with remdesivir, an antiviral medication, when compared with placebo ^30^. The TACTIC-R (NCT04390464) and TACTIC-E (NCT04393246) trials are investigating repurposed and experimental immunomodulatory medications, respectively, for COVID-19 across the UK. In addition, the PRINCIPLE trial (ISRCTN86534580) has demonstrated that the MAMS platform can also be adopted for assessment of potential interventions in a primary care setting during a global health pandemic.

Whilst this article has focused on the MAMS platform approach, additional and complementary multi-arm approaches can be used, including multi-arm, multi-factorial designs - such as the REMAP-CAP trial (NCT02735707). Notably, factorial approaches can also be combined with MAMS designs. A multi-arm, multi-factorial design may offer particular benefits when assessing multiple combinations of interventions, as is typically the case for patients being treated in an intensive care environment. Regardless of the exact adaptive platform trial design selected, given the complexities, costs and workload associated with delivering complex innovative trials ^31– 33^, especially in the late-phase trial setting, these platform trials are likely best delivered by multi-disciplinary organisations and teams with experience and track record for success.

## Challenges of a MAMS platform approach for pandemic trials

An often highlighted challenge, especially for longer-lasting platform protocols, is the potential for standard-of-care to change during the course of the trial and implications of this for a MAMS design ^31^. Indeed, in the context of a fast-moving infectious disease pandemic, it is likely that usual or standard care will change based on interim analysis of comparison arms (and reporting) from the current trial, or from emerging data and findings of other research studies. However, there are now well-reported methods to overcome the challenges from changing standards of care in a MAMS design ^34^. These solutions principally ensure comparison of interventions to participants recruited to a contemporary standard-of-care treatment arm, rather than comparison with participants receiving historical standard-of-care treatment. With respect to reporting of results in a MAMS trial, comparison arms will have primary results become available at different times during the course of the platform. Accordingly, primary outcome results from intervention arms should be reported as soon as these results have been analysed and verified. Indeed, in a pandemic setting it would likely be unethical to withhold or delay reporting of primary results from comparison arms, particularly when such findings will provide evidence for a better standard of care. Given the length of time and delays in traditional peer review processes, an important action noted during the COVID-19 pandemic, has been a move by trial teams towards earlier dissemination of findings such as through the use of open-access, pre-print reports ^35, 36^.

When adding new interventions to a MAMS platform, consideration of type one error rates (i.e. possibility for greater false positive findings for interventions in these trials) is important ^37^. In this regard, for MAMS platforms, early engagement is advised by trial teams with healthcare regulators to discuss issues about multiplicity and requirements for clinical trial authorisation. In addition, a particular complexity of trials within a pandemic is the typically limited information about a disease and equally limited information known about the efficacy of potential interventions. Therefore, it may not be desirable to commit to a specific sample size, as would occur in conventional RCTs, until a reasonable amount of data is observed within the trial. Indeed, this is well illustrated by the RECOVERY trial, where there was no initial sample size estimation. However, based on data accrual and events of interest, subsequent sample size calculations could be made.

Setting up and undertaking multi-national trials of any design in non-pandemic settings can offer considerable challenges ^38^, and the difficulties may be an order of magnitude greater in a pandemic - given the speed needed to setup these trials. Challenges to consider include ensuring adequate funding, staffing, alignment of a single trial protocol to enable appropriate regulation, monitoring and oversight in each country, and logistical considerations, such as implementation of protocol amendments simultaneously across participating countries and sites. An additional challenge, which can be very difficult to predict, will be which countries are most likely to be affected by a pandemic and at what time they are most likely to be affected. The relative national incidence of a disease, and fluctuations in these numbers, will have a critical bearing on the ability to recruit into any pandemic trial. Moreover, different nations will have variable infrastructures in place to allow rapid setup of sites and ability to deliver RCTs efficiently, including crucial aspects such as rapid distribution of medications. The MAMS platform approach requires great consideration and planning. Therefore, a key challenge to overcome for greater delivery and use of this design is to ensure collaboration between individuals and organisations with experience in delivering such trials. In this respect, there are recent commendable international collaborative efforts to combine expertise and experience from across academia and industry, including the European union - patient-centric clinical trial platforms (EU-PEARL) consortium and the clinical trials transformation initiative (CTTI).

It is sobering to reflect that a likely major contributor to the speed of setup for RECOVERY was that this trial was taking place in one country and within one healthcare system - without the need to overcome multiple regulatory and administrative hurdles across multiple countries. Moreover, realistically it is only feasible for a few such large, platform trials to be supported by local, regional and national research infrastructures – therefore collaborative working from the outset and speed to setup a MAMS platform are crucial in a pandemic setting. The WHO SOLIDARITY trial approach likely offers the best practical solution around some of these challenges for multi-national trials – using a single overarching master protocol, with individual and separate trial registrations and delivery in each respective country.

## Future horizons

An important future consideration is the need to collate and synthesise information from RCTs in order to derive maximum benefit for patients worldwide. Accordingly, there is an urgent need for collaboration between trial teams, to ensure the most accurate and rapid reporting of findings.

This collaboration could be achieved using the prospective framework for adaptive meta-analysis (FAME), which has been successfully used to pool aggregate data across ongoing trials of prostate cancer ^39, 40^, and helped facilitate the identification of patient subgroups for whom individual treatments may be most effective ^41^. We note recent commendable findings from the WHO rapid evidence appraisal for COVID-19 therapies (REACT) working group - reporting steroid use across seven COVID-19 RCTs and demonstrating consistent findings of benefit in hospitalised patients, especially those requiring supplemental oxygenation or respiratory support ^42^. In the COVID 19 setting, meta-analysis using individual participant data (IPD) may be key to delineating which treatment strategies are most effective in individual patients ^43^. Irrespective of which method is used, extending the FAME approach to include prospective, collaborative agreements to share data from relevant trials enables rapid synthesis and reporting of all the evidence, providing a clear message to patients, clinicians and the wider public. 

It is also clear that there is a need to be prepared for both further waves of COVID-19 and future potential global health pandemics. A key aspect of preparedness will be for trial organisations to pre-prepare MAMS protocols ready to implement at speed, ideally using a seamless phase two/three approach. Given the multiple potential vaccines under development for COVID-19 ^44^, appropriate consideration should be given to the application of MAMS designs to preventative vaccine trials in the near future. Similarly to treatment trials, the WHO is commendably supporting global efforts for evaluation of multiple vaccines in a SOLIDARITY vaccine trial protocol ^45^.

## Conclusion

There is an urgent need for reliable evidence in pandemic settings, as illustrated by the COVID-19 pandemic. Well conducted, adaptive platform trials utilising a MAMS approach, offer substantial advantages over multiple, separate, two-arm trials. Indeed, trials using efficient approaches have provided some of the rapid answers needed in the COVID-19 pandemic. However, these efficient trial approaches have been the exception rather than the rule. In this respect, an important consideration for the future will be for funders, regulators and other key stakeholders to prioritise more efficient trial designs. Focusing efforts on MAMS protocols in particular, with international collaboration between co-ordinating trial teams, and prospectively planned meta-analyses of emerging data, should lead to faster identification of effective therapies and vaccines. This should also contribute to faster answers for patients, clinicians, and health care systems around the world, including for further waves of COVID-19 and enable preparedness for future global health pandemics.

## Data availability

No data is associated with this article.
