# Supplementary material for: Adaptive platform trials using multi-arm, multi-stage protocols: getting fast answers in pandemic settings
Source: F1000Res. 2020 Nov 23;9:1109. Originally published 2020 Sep 9. [Version 2] doi: 10.12688/f1000research.26253.2 (PMC7596806; doi:10.12688/f1000research.26253.2)
Supplement: Peer review history

# Peer review: Reviewer response for version 1

**DOI:** 10.5256/f1000research.28976.r73671

**Published:** 2020-10-29

**Authors:** Dmitri Nepogodiev

**Affiliations:** 1 University of Birmingham, Birmingham, UK

The authors make a clear case for the benefits of adaptive platform trials in the context of the COVID-19 pandemic. The RECOVERY trial has demonstrated that MAMS design can significantly accelerate the rate at which urgent research questions can be robustly answered. However, even with the increased efficiency that MAMS offers, such platform trials still require large numbers of patients to be recruited. For example, RECOVERY has now recruited 15,000 patients but this has required concerted recruitment across most (n=176) UK hospitals. Realistically, during a pandemic it is only feasible for available local research infrastructure to support a limited number of such large-scale trials. This raises issues about how a consensus is reached on what trials are supported and what treatments they test.

1. What research questions should be addressed? There are a large number of research questions relating to SARS-CoV-2 across different domains (prophylaxis, treatment, public health measures etc), different patient groups, and different settings (primary care, acute hospitals, nursing homes etc). It is not feasible to support a large number of trials so research questions must be prioritised. Who should do this and on what basis?
2. Who should lead such trials? This requires multi-disciplinary (multi-institutional) teams with a track record of delivering large-scale studies. A pandemic is probably not the best time for novice trialists to get started.
3. For a treatment trial (e.g. RECOVERY) how should treatments be selected? Although a large number of potential repurposed treatments were proposed, there was relatively little early phase research to indicate which should go to phase III trials. So who should make these decisions and how?
4. New treatments are now being developed for COVID-19. How should a pipeline be set up linking phase II studies to the phase III platform trial to ensure that promising treatments seamlessly and efficiently progress?
5. Once the results for a trial arm have been analysed and final results are available how should the results be disseminated? Should this be through the traditional peer review, pre-print servers, press release? How can research findings be rapidly linked in to clinical guidelines?

Although these points are beyond the specifics of the MAMS design, they are important to the overall aim of rapidly delivering impactful research during the pandemic and they are likely to be issues faced by anyone setting up a MAMS trial in this context. Perhaps a few brief comments on this might be interesting?

Is the topic of the opinion article discussed accurately in the context of the current literature?

Yes

Are arguments sufficiently supported by evidence from the published literature?

Yes

Are all factual statements correct and adequately supported by citations?

Yes

Are the conclusions drawn balanced and justified on the basis of the presented arguments?

Yes

Reviewer Expertise:

Public health; epidemiology; surgery

I confirm that I have read this submission and believe that I have an appropriate level of expertise to confirm that it is of an acceptable scientific standard.

# Response

**Published:** 2020-11-17

**Authors:** Nurulamin Noor

**Affiliations:** Medical Research Council Clinical Trials Unit at University College London, UK

We are grateful to both reviewers for their careful assessment of this manuscript, and their constructive comments.

 We have provided a point-by-point response to each of the reviewers’ comments below, added in citations to support the additional text, and where applicable updated current citations. The changes to the manuscript are described and, where applicable, added changes are highlighted as tracked changes in the revised manuscript.

 Best wishes,

**Reviewer: 2**

 Comment: The authors make a clear case for the benefits of adaptive platform trials in the context of the COVID-19 pandemic. The RECOVERY trial has demonstrated that MAMS design can significantly accelerate the rate at which urgent research questions can be robustly answered.

 Response: Thank you for your positive comments and for your careful consideration of this manuscript.

 Comment: However, even with the increased efficiency that MAMS offers, such platform trials still require large numbers of patients to be recruited. For example, RECOVERY has now recruited 15,000 patients but this has required concerted recruitment across most (n=176) UK hospitals. Realistically, during a pandemic it is only feasible for available local research infrastructure to support a limited number of such large-scale trials. This raises issues about how a consensus is reached on what trials are supported and what treatments they test.

 Response: Thank you for this question about prioritisation of trials in a pandemic. We agree that one of the benefits from using the MAMS platform protocol approach is the reduced competition between trials and greater efficiencies overall. We also agree that another source of efficiency will be from approaches where there are wide-scale support for fewer platforms rather than many overlapping and competing platforms. Ultimately, in a pandemic setting given the need for speed for answers, we believe the platforms, which can be setup at speed to start answering research questions are most likely to provide the answers needed. We have now included these above points in our discussion.

 Comment: What research questions should be addressed? There are a large number of research questions relating to SARS-CoV-2 across different domains (prophylaxis, treatment, public health measures etc), different patient groups, and different settings (primary care, acute hospitals, nursing homes etc). It is not feasible to support a large number of trials so research questions must be prioritised. Who should do this and on what basis?

 Comment: For a treatment trial (e.g. RECOVERY) how should treatments be selected? Although a large number of potential repurposed treatments were proposed, there was relatively little early phase research to indicate which should go to phase III trials. So who should make these decisions and how?

 Comment: New treatments are now being developed for COVID-19. How should a pipeline be set up linking phase II studies to the phase III platform trial to ensure that promising treatments seamlessly and efficiently progress?

 Response: Thank you for these three linked questions about selection of research questions and interventions within a MAMS platform protocol. We entirely agree that the MAMS platform can be used to evaluate a number of different interventions. To date, the majority of interventions that have been assessed have been investigational medicinal products (IMPs) for treatment. However, we strongly believe that non-IMP interventions also require assessment in a pandemic setting. The MAMS platform offers many benefits for evaluating prophylactic, treatment or preventative interventions. Ultimately the decision for which interventions should be studied within a trial platform including future intervention arms, rest with the trial team itself. We have now included this in the discussion and about factors for trial teams to consider when adding in new intervention arms and assessing their progress. 

 Comment: Who should lead such trials? This requires multi-disciplinary (multi-institutional) teams with a track record of delivering large-scale studies. A pandemic is probably not the best time for novice trialists to get started.

 Response: We thank the reviewer for this question and agree that given the complexities and workload associated with delivering MAMS platform trials, especially in the late-phase trial setting, these trials would likely be best delivered by multi-disciplinary organisations and teams with experience and track record in the area. We have included this in our discussion section and cited three citations further exploring MAMS platform trials from: a trial management perspective (Schiavone et al. Trials, 2019), a data management perspective (Hague et al. Trials, 2019), and from a trial team perspective for working within and delivering large MAMS platform trials in the late-phase trial setting (Morrell L et al. Trials, 2019).

 Comment: Once the results for a trial arm have been analysed and final results are available how should the results be disseminated? Should this be through the traditional peer review, pre-print servers, press release? How can research findings be rapidly linked in to clinical guidelines? Although these points are beyond the specifics of the MAMS design, they are important to the overall aim of rapidly delivering impactful research during the pandemic and they are likely to be issues faced by anyone setting up a MAMS trial in this context. Perhaps a few brief comments on this might be interesting?

 Response: Thank you for this question about the important topic of when and how to report findings from comparison intervention arms during the course of a MAMS platform. The challenge of rapid and appropriate dissemination is pertinent to all trials. In a MAMS platform trial where comparison arms will have primary results available at different times during the course of the platform, we strongly believe that primary outcome results from intervention arms should be reported as soon as these results have been analysed and are available - and that it would be unethical to delay reporting these findings until all comparison arms have primary results available. We support the process of traditional peer review but given the length of time that this can take, we also support appropriate use for earlier dissemination of headline findings such as through the use of open-access, pre-print reports. We have now included a section on this topic in the challenges section of our manuscript.

# Peer review: Reviewer response for version 1

**DOI:** 10.5256/f1000research.28976.r71099

**Published:** 2020-10-08

**Authors:** Cyrus Mehta

**Affiliations:** 1 Cytel Corportation, Cambridge, MA, USA

**General Comments**

 This paper highlights the many benefits of designing a randomized clinical trial so that several treatment arms are compared in a pairwise fashion to a common control arm, with interim analyses to identify effective treatments and drop ineffective ones from further consideration. The considerable efficiency that such a design offers relative to the more conventional two-arm clinical trial arises in three ways. First, since many treatment arms share the same control arm, there is a saving of sample size. Second, the efficacy and/or futility data for each of the treatment interventions is obtained much earlier, and more reliably, than it would be were the interventions to be studied in separate two-arm trials that are conducted in sequence and with smaller sample sizes. Thirdly the operational infrastructure associated with setting up the multi-arm trial need only be created once, and can be used repeatedly, with minor adjustments, as current treatment arm exit the platform and new arms replace them. The references to on-going and completed trials, many of which are pandemic related, are a special strength of this paper.

 The authors have also pointed some of the challenges of the MAMS platform approach. Much of this discussion relates to aligning the special requirements of participants from different countries and sites, with different medical needs, different treatment interventions and different funding sources and onto a single protocol. This is a vast problem that cannot be discussed adequately in a short survey paper. Perhaps it is worthwhile to mention in this context the EU-PEARL Consortium (eu-pearl.eu).

**Issues in Need of Further Discussion:**

1. **Changing the Control Arm**. How does one handle the problem of comparability if one of the interventions attains statistical and clinical significance at an interim analysis and thereby becomes the new control arm? Suppose, for example, that while subjects are still being followed, one of the arms attains statistical significance by crossing a group sequential boundary. Would you continue with the current SOC until the pre-specified follow-up of the remaining arms was completed? Would that be ethical? On the other hand, if you were to immediately replace the SOC with the new intervention, how would you interpret the results of a comparison to a control arm that was replaced in mid-stream?
2. **Concurrent Controls.** If one or more arms are dropped for futility and are replaced by new arms, what are your thoughts on using all the control-arm data in the comparison with the new arms, versus using only the concurrent control-arm data? Some Bayesian trials have used the former approach.
3. **Control of Multiplicity.** Multiplicity issues can arise if one performs repeated significance tests over time on a specific treatment arm to determine if it is efficacious. This source of multiplicity can be controlled by use of group-sequential efficacy boundaries. A different source of multiplicity arises because multiple treatments are being tested in the same protocol. Therefore one must ask whether these treatments are members of one family or whether there is no relationship whatsoever between them, and they have merely been placed in the same protocol for the convenience of sharing a common control arm. In the former case the family-wise error rate must be controlled while in the latter case, only the pair-wise error rate must be controlled. Which type of control is more appropriate in the Covid setting? These are important conceptual issues that should be discussed.
4. **Adaptive Sample Size Re-assessment.** What are the authors' views on estimating the treatment effect at an interim analysis and adjusting the sample size based on the new evidence from the trial itself? Appropriate statistical methods are available to control the error rate in this situation. In a Covid setting, where very little is known about the efficacy of the treatment interventions, would it not be desirable to commit to a final sample size only after having observed a reasonable amount of data from the trial?

Is the topic of the opinion article discussed accurately in the context of the current literature?

Yes

Are arguments sufficiently supported by evidence from the published literature?

Yes

Are all factual statements correct and adequately supported by citations?

Yes

Are the conclusions drawn balanced and justified on the basis of the presented arguments?

Yes

Reviewer Expertise:

I am a biostatistician with expertise in design of adaptive clinical trials

I confirm that I have read this submission and believe that I have an appropriate level of expertise to confirm that it is of an acceptable scientific standard.

# Response

**Published:** 2020-11-17

**Authors:** Nurulamin Noor

**Affiliations:** Medical Research Council Clinical Trials Unit at University College London, UK

We are grateful to both reviewers for their careful assessment of this manuscript, and their constructive comments.

 We have provided a point-by-point response to each of the reviewers’ comments below, added in citations to support the additional text, and where applicable updated current citations. The changes to the manuscript are described and, where applicable, added changes are highlighted as tracked changes in the revised manuscript.

 Best wishes,

**Reviewer: 1**

 Comment: This paper highlights the many benefits of designing a randomized clinical trial so that several treatment arms are compared in a pairwise fashion to a common control arm, with interim analyses to identify effective treatments and drop ineffective ones from further consideration. The considerable efficiency that such a design offers relative to the more conventional two-arm clinical trial arises in three ways. First, since many treatment arms share the same control arm, there is a saving of sample size. Second, the efficacy and/or futility data for each of the treatment interventions is obtained much earlier, and more reliably, than it would be were the interventions to be studied in separate two-arm trials that are conducted in sequence and with smaller sample sizes. Thirdly the operational infrastructure associated with setting up the multi-arm trial need only be created once, and can be used repeatedly, with minor adjustments, as current treatment arm exit the platform and new arms replace them. The references to on-going and completed trials, many of which are pandemic related, are a special strength of this paper.

 Response: Thank you for your positive comments and for your careful reading of this manuscript.

 Comment: The authors have also pointed some of the challenges of the MAMS platform approach. Much of this discussion relates to aligning the special requirements of participants from different countries and sites, with different medical needs, different treatment interventions and different funding sources and onto a single protocol. This is a vast problem that cannot be discussed adequately in a short survey paper. Perhaps it is worthwhile to mention in this context the EU-PEARL Consortium (eu-pearl.eu).

 Response: We thank the reviewer for this suggestion and agree that these are important points that will likely be best considered by international collaborative efforts. We have now included reference to important international efforts in this regard, including mention of both the EU-PEARL Consortium and to the Clinical Trials Transformation Initiative (CTTI).

 Comment: Changing the Control Arm. How does one handle the problem of comparability if one of the interventions attains statistical and clinical significance at an interim analysis and thereby becomes the new control arm? Suppose, for example, that while subjects are still being followed, one of the arms attains statistical significance by crossing a group sequential boundary. Would you continue with the current SOC until the pre-specified follow-up of the remaining arms was completed? Would that be ethical? On the other hand, if you were to immediately replace the SOC with the new intervention, how would you interpret the results of a comparison to a control arm that was replaced in mid-stream?

 Response: Thank you for this question about change in standard of care for control arms. We certainly agree that in the context of a fast-moving infectious disease pandemic, it is likely that usual or standard care will change based on interim analysis (and reporting) of comparison arms. We agree that it would be unethical to continue treating new patients with a historical standard of care in light of an evidence-based and available better standard of care, and have now made this clear in the discussion. We have also included about the need to compare comparison/intervention arms in a pairwise manner with the participants in the standard of care arm, at the time of enrolment. For ongoing patients in the trial when convincing results emerge, there would need to be careful consideration for each participant – the trial results apply to patients who meet the entry criteria and many ongoing participants may no longer be in this same state; this is more likely in trials and disease courses that run over a longer period.

 Comment: Concurrent Controls. If one or more arms are dropped for futility and are replaced by new arms, what are your thoughts on using all the control-arm data in the comparison with the new arms, versus using only the concurrent control-arm data? Some Bayesian trials have used the former approach.

 Response: Thank you for this challenging question on use of control data from trials. We agree that there has been variable practice across platform trials. Whilst some trials do use historical control group data from the entirety of a platform trial, we encourage applying caution, particularly in the late-phase trial setting, and when there a changes in standard of care for the control arm group within the course of a trial. Staying with the contemporaneously randomised patients is likely the better course of action in most instances.

 Comment: Control of Multiplicity. Multiplicity issues can arise if one performs repeated significance tests over time on a specific treatment arm to determine if it is efficacious. This source of multiplicity can be controlled by use of group-sequential efficacy boundaries. A different source of multiplicity arises because multiple treatments are being tested in the same protocol. Therefore one must ask whether these treatments are members of one family or whether there is no relationship whatsoever between them, and they have merely been placed in the same protocol for the convenience of sharing a common control arm. In the former case the family-wise error rate must be controlled while in the latter case, only the pair-wise error rate must be controlled. Which type of control is more appropriate in the Covid setting? These are important conceptual issues that should be discussed.

 Response: Thank you for this question about multiplicity, which is of interest to many of the authors. We have now included a short discussion about multiplicity and control of error rates when adding intervention arms in the context of a pandemic platform and added in a recent citation to support this text (Choodari-Oskooei et al. Clinical Trials, 2020).

 Comment: Adaptive Sample Size Re-assessment. What are the authors' views on estimating the treatment effect at an interim analysis and adjusting the sample size based on the new evidence from the trial itself? Appropriate statistical methods are available to control the error rate in this situation. In a Covid setting, where very little is known about the efficacy of the treatment interventions, would it not be desirable to commit to a final sample size only after having observed a reasonable amount of data from the trial?

 Response: Carefully planned and explained adaptive sample size re-assessment could also be incorporated into a MAMS platform protocol. However, this is a controversial topic. We do advise an element of caution as with all adaptations made following interim analysis in terms of clarifying which individuals/committees have access to unblinded interim outcome data and pre-specifying how data will be used to inform decision-making. We note with interest that the RECOVERY trial - used as one of the exemplar trials in this manuscript - did not have an initial sample size calculation, and used data from early periods within the trial to allow subsequent sample size calculations. We have now accommodated this in the discussion section of the manuscript.
